# The Society of Homœopathicians

**Published:** 1895-01

**Authors:** Clarence Willard Butler

**Affiliations:** Montclair, N. J.


### THE SOCIETY OF HOMŒOPATHICIANS.

CLARENCE WILLARD BUTLER, M. D., MONTCLAIR, N. J.

In the manifesto of the new Homœopathic Association with the above title, published in THE HOMŒOPATHIC PHYSICIAN for November, 1894, appears the following:

"At the meeting held at Narragansett Pier, June 21st, 1892, the I. H. A. deliberately violated its Declaration of Principles, Constitution, and By-laws, by voting to indefinitely postpone a report of the Board of Censors before it had been submitted, in order to shield a member in error. This repudiation of its principles destroyed the life of pure Homœopathy as represented by the I. H. A., and no 'resolution' can restore that life or the integrity of the Association."

The central fact in respect of this matter has been concisely stated by the writer of the quoted paragraph. It is true that the I. H. A. did vote "to indefinitely postpone a report of the Board of Censors before it had been submitted," but that such action was deliberate; that it was done to shield a member in error; that it was a violation of its Declaration of Principles, Constitution, and By-laws; that it destroyed the "life of pure Homœopathy as represented by the I. H. A.;" that it destroyed the integrity of the Association—all seem assumptions pure and simple, and without justification in the circumstances immediately surrounding the event, or in subsequent developments in relation to it.

Now, the Board of Censors not only had a right to present its report, but it was its manifest duty to do so. By refusing it opportunity to exercise this right and to perform this function, the Association acted contrary to the customs and usages of all deliberative bodies, and without doubt a ready and acute parliamentarian would have been able to prevent its unwise action. It violated the customs and usages of deliberative bodies—that is, it violated parliamentary law. But pray, how did it violate *its* Declaration of Principles and *its* Constitution? Inasmuch as it is usually expressed or assumed by the By-Laws of a society that its proceedings shall be conducted according to parliamentary usage, it may be said that it, by unparliamentary action, indirectly violated its By-Laws perhaps; but surely as far as the objects of the Association are concerned, its principles in relation to that branch of science which is its especial business, or any other matter aside from the manner in which its deliberations shall be conducted, parliamentary law has nothing whatever to do. That, in the specific case under consideration, the violation of this law was a grave and serious error, there can be no doubt. That it established a precedent of dangerous import, offered a gratuitous and wholly undeserved affront to its Board of Censors, and tended to subversion of orderly and business-like methods, one may readily concede and sincerely regret.

But on what possible grounds it can be assumed that it violated the Declaration of Principles of the Society, its Constitution, or even otherwise that indirectly its By-Laws, it is difficult to conceive.

And it is equally difficult to understand why the new society charges the I. H. A. with *deliberate* action on this occasion. Had it been the deliberate intention of the Association, or a majority of its members to suppress the report of the Board of Censors, it would have carefully avoided infringements of law and leave no loop-hole for future criticisms of its methods, even if unable to prevent strictures upon its intent.

That it was the deliberate judgment of the majority that no good could come from discussion of the charges made, and its deliberate intention, not only to prevent such discussion but to ignore the charges, seems more than probable. And had the Association proceeded in an orderly manner to carry out the desires of a majority of its members in this regard, whatever may be the opinion held of the wisdom or unwisdom of such a course of action, there can be no question but that it had an undoubted right to take this course.

The very fact that it did not proceed in an orderly manner according to parliamentary usage to accomplish its probable desires, shows how precipitate and unthinking its action undoubtedly was. That this ill-considered and hasty action was for the purpose of shielding "a member in error" seems also pure assumption. The charges were informally before the Association, and the reply to the charges had, also in an informal manner, been submitted to its members. It seems to me more reasonable to assume that the members were disposed to believe that the charges could not be sustained and were anxious to avoid the heat and heart-burnings of open discussion. Had the new society chosen to criticise the wisdom of this course, probably now no one would arise to say them "nay," but to thus openly assume that the I. H. A. deliberately chose to shield error, rather than to expose and condemn it, seems, especially in respect of unproven charges, as unjust as it is uncharitable. Neither is "the integrity of the I. H. A." nor the "life of pure Homœopathy as represented in it" dead. It is prosperous, virile, and aggressive, and the enthusiasm for the Homœopathy of Hahnemann, the determination to maintain and advance it, the indications that no errors of the past shall hamper its future, are all "in evidence" among its members.

Whether "a new society is imperative for the preservation and advancement of pure Homœopathy" is a question to which perhaps no answer may now be given. Assuredly there is ample room for all, and much work in sight.

How this shall best be done, who can tell?

> "God deigns not to discuss
> With our impatient and o'erweening wills
> His times, and ways of working out through us
> Heaven's slow but sure redress of human ills."

